# Germ cells in the teleost fish medaka have an inherent feminizing effect

**DOI:** 10.1371/journal.pgen.1007259

**Published:** 2018-03-29

**Authors:** Toshiya Nishimura, Kazuki Yamada, Chika Fujimori, Mariko Kikuchi, Toshihiro Kawasaki, Kellee R. Siegfried, Noriyoshi Sakai, Minoru Tanaka

**Affiliations:** 1 Division of Biological Science, Nagoya University, Nagoya, Aichi, Japan; 2 Genetic Strains Research Center, National Institute of Genetics, Mishima, Shizuoka, Japan; 3 Department of Genetics, SOKENDAI (The Graduate University for Advanced Studies), Mishima, Shizuoka, Japan; 4 Biology Department, University of Massachusetts Boston, Boston, Massachusetts, United States of America; University of Wuerzburg, GERMANY

## Abstract

Germ cells give rise to eggs or sperm. However, recent analyses in medaka (*Oryzias latipes*) showed that germ cells are also important for feminization of gonads, although this novel role of germ cells has not been characterized in detail. Here, we show that the feminizing effect is inherent to germ cells and is not affected by gametogenic stages or the sexual fate of germ cells. Three medaka mutants were generated to demonstrate this effect: *figlα* mutants, in which follicle formation is disrupted; *meioC* mutants, in which germ cells are unable to commit to gametogenesis and meiosis; and *dazl* mutants, in which germ cells do not develop into gonocytes. All these different stages of germ cells in XX mutants have an ability to feminize the gonads, resulting in the formation of gonads with ovarian structures. In addition to normal ovarian development, we also suggest that the increased number of gonocytes is sufficient for male to female sex reversal in XY medaka. These results may genetically demonstrate that the mechanism underlying the feminizing effect of germ cells is activated before the sexual fate decision of germ cells and meiosis, probably by the time of gonocyte formation in medaka.

**Author summary:**

Germ cells are the only cells that can transfer genetic materials to the next generation via the sperm or egg. However, recent analyses in teleosts revealed another essential role of germ cells: feminizing the gonads. In our study, medaka mutants in which gametogenesis was blocked at specific stages provides the novel view that the feminizing effect of germ cells occurs in parallel with other reproductive elements, such as meiosis, the sexual fate decision of germ cells, and gametogenesis. Germ cells in medaka may have a potential to feminize gonads at the moment they have developed.

## Introduction

The sex of organisms is determined either by genetic factors and/or by environmental factors. Many organisms with genetic sex determination systems have sex determination genes, but these genes differ from species to species [1]. The environmental factors that determine sex include temperature, density, pH and social status [2]. Although initial triggers for sex determination vary, there are some common features in the subsequent sex differentiation process in gonads. One feature is observed in the sexual modes of germ cell proliferation. In most vertebrates, after primordial germ cells (PGCs) reach the gonads, PGCs develop to become gametogenesis-competent germ cells [3]. Whereas the germ cells in females begin to differentiate to undergo oogenesis, the germ cells in males are in a mitotically quiescent state for a while before the initiation of spermatogenesis [4, 5].

The teleost fish, medaka (*Oryzias latipes*), is one of the organisms in which sex is determined genetically [6, 7]. The sex determination gene on the medaka Y chromosome, *DMY*/*dmrt1bY*, is initially expressed in the gonadal somatic cells when the gonads form at stage 33 [4 day-post-fertilization (dpf)] [8]. At this stage, the sexual difference in the number and differentiation of germ cells is not observed. The first appearance of sexual differentiation in the gonad can be recognized by the time of hatching [0 day-post-hatching (dph)][9]. Germ cells in XX gonads initiate oogenesis to enter meiosis around the time of hatching, or several days after hatching, while proliferation of germ cells and initiation of gametogenesis is suppressed in XY germ cells during the larval stage. Spermatogenesis is initiated approximately one month after hatching [10].

Previously, we found that the occurrence of more germ cells in female medaka gonads is not just a result of sex differentiation, but is essential for female differentiation of gonads in medaka. When germ cells are ablated in medaka, XX fish show female-to-male sex reversal, while XY fish with overproliferated germ cells, which is usually associated with oogenesis, exhibit male-to-female sex reversal [11–13]. In support of this notion, studies have reported that high temperatures also decrease the number of germ cells, which often accompanies female-to-male sex reversal [14, 15]. Therefore, although the sex of medaka is genetically determined by *DMY*/*dmrt1bY*, the germ cells seem to play a critical role in sex determination, especially in the feminization of gonads. This finding implies that, in addition to the most well-known role of germ cells to become gametes, germ cells have a unique and surprising potential to change their surrounding environment. However, the molecular basis of, and the stage of gametogenesis critical for feminization remain unknown.

It has been demonstrated that germ cells in the gonads have to acquire competence for gametogenesis before and/or at the time of entering the gonadal area [3, 16]. In medaka, gametogenesis-competent cells in the gonad can be categorized into two types, type I and type II, which are distinguished by the modes of cell division [9]. Type I cells are characterized by stem-type self-renewal division and are histologically defined as gonocytes in the developing gonads. They are completely enclosed by supporting cells and are present in isolation. Certain populations of type I germ cells commit to gametogenesis and undergo type II division, in which germ cells connected by the intercellular bridge synchronously divide to form a cyst. After undergoing type II division, germ cells enter meiosis and initiate folliculogenesis or spermatogenesis [10].

In this study, medaka mutants with the disrupted gene, *figlα*, *meioC* or *dazl*, were used to determine a critical gametogenetic stage(s) for the feminization. In addition, *gsdf* medaka mutant was used to study the requirements of germ cells in the process of male-to-female sex reversal. Collectively, we suggest that germ cells in medaka are predisposed to develop the ability to feminize the gonad independent of somatic sex. The feminizing effect of germ cells seems to be underlain by a mechanism that is distinct from the mechanisms of commitment to gametogenenesis, entering meiosis and sexual fate determination.

## Results

### Ovarian follicles are not required for ovary fate and female development in medaka

After cystic division and the start of meiosis, each oocyte at diplotene stage is surrounded by somatic cells (granulosa cells and theca cells) to form follicles in female gonads. We initially assumed that follicles are essential for the feminization of gonads in medaka for three reasons. First, the somatic cells of follicles consist of granulosa cells expressing *foxl2* and theca cells expressing *aromatase* [17, 18]. Both genes are involved in feminization. Second, in male-to-female sex reversal due to overproliferation of germ cells in medaka, follicle formation is observed during early gonadal development [12, 13, 19]. Third, in zebrafish, follicles are considered to be essential for the feminization of gonads [20, 21]. Therefore, we first generated mutant medaka in which follicle formation was disrupted.

**Fig 1:**
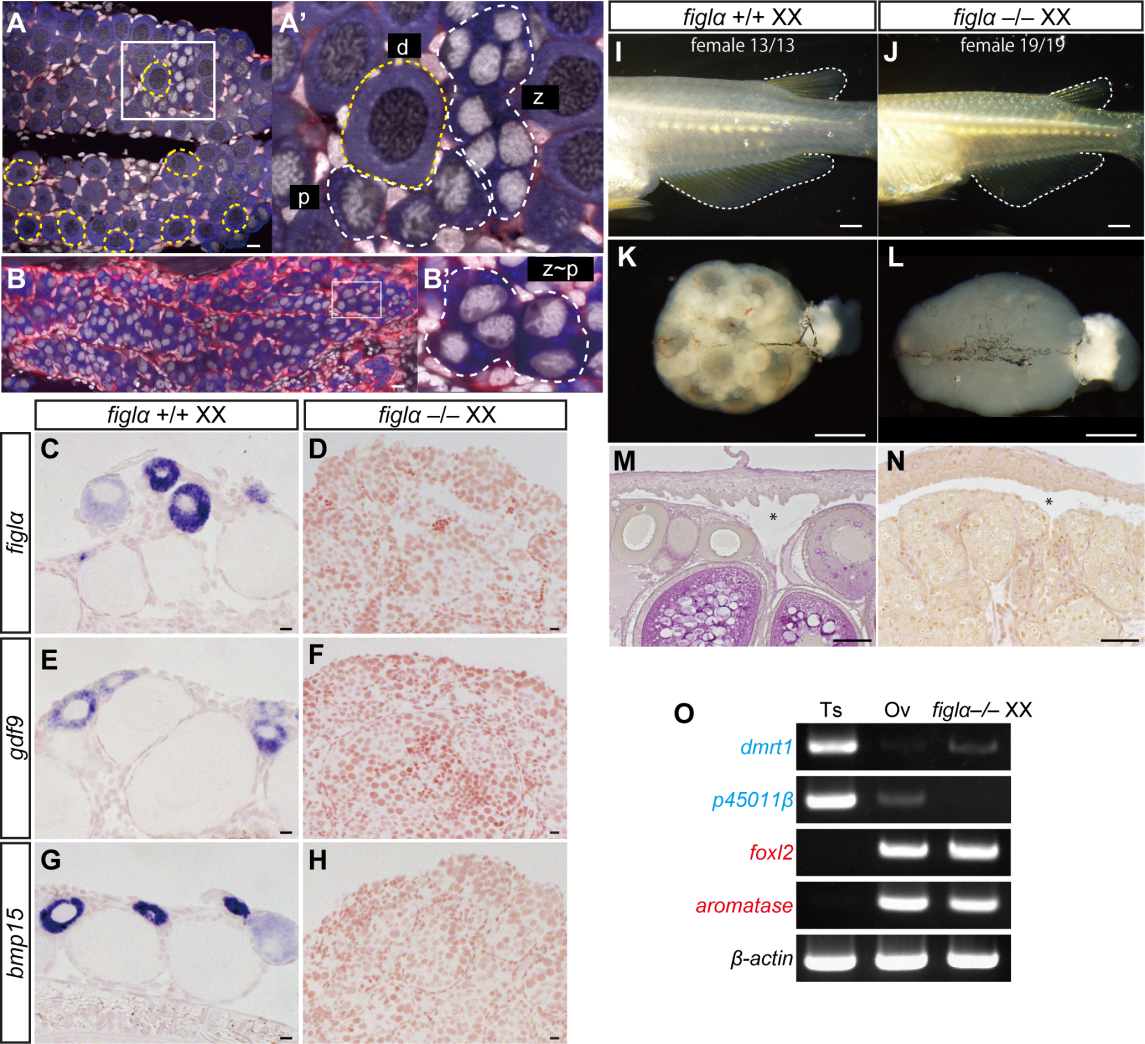
Follicles are not required for normal female development. (A) *figlα*+/+ XX gonads at 10 dph. The inset is magnified in (A’). By 10 dph, meiotic germ cells at zygotene (z), pachytene (p), and diplotene (d) stages are present in control XX gonads. Yellow dotted-lines encircle follicles. (B) *figlα*–/–XX gonads at 10 dph. The inset is magnified in (B’). Germ cells beyond pachytene stage are not observed in the mutant gonads. Blue: OLVAS (germ cell), red: *sox9b*-DsRed (supporting cells), gray: DAPI (nucleus). (C–H) *In situ* hybridization for *figlα* and the follicle marker genes *gdf9* and *bmp15* using adult gonads. In *figlα*+/+ control ovaries, *figlα*, *gdf9*, and *bmp15* were predominantly expressed in the diplotene oocytes (purple signals, C, E, and G). In *figlα*–/–XX gonads, these transcripts were not detected (D, F, H). (I and J) The shapes of the dorsal and anal fins of medaka constitute the secondary sex characteristics. Round fins are characteristic of the female. Thirteen of 13 (13/13) XX *figlα*+/+ and 19/19 of XX *figlα*–/–mutants were female. (K and L) External appearance of a control ovary (K) and a *figlα*–/–gonad (L). (M and N) Cross sections of adult gonads with PAS staining. Asterisks indicate the ovarian cavity, showing the typical structure of the ovaries. (O) RT-PCR analysis of male markers, *dmrt1* and *p45011β*, and female makers, *foxl2* and *aromatase*, in adult gonads. The expression pattern of *figlα*–/–XX gonads was similar to that of control ovaries. Scale bars are 10 μm (A–H), 1 mm (I–L) and 50 μm (M and N).

*Figlα (**F**unction* *i**n* *g**erm* *l**ine* *a**lpha)* is a basic helix-loop-helix transcription factor that is essential for follicle development in mammals [22, 23] and is preserved in other vertebrates including teleosts [24, 25]. In medaka, *figlα* transcripts were detected in female germ cells (S1A and S1C Fig) but not in male germ cells at 10 days post hatching (dph) and adult stage (S1B and S1D Fig). Detailed analysis of *figlα* expression was performed using *figlα*-EGFP reporter transgenic medaka, in which EGFP was expressed under control of *figlα* regulatory elements (S1E Fig). *Figlα*-EGFP was initially detected in germ cells at the late zygotene stage, and strong *figlα*-EGFP signals were detected in diplotene oocytes (S1F Fig), suggesting that *figlα* functions in germ cells after entering meiosis, possibly for the formation of follicles. To determine whether deletion of the *figlα* gene disrupts follicle formation in medaka, we generated a medaka *figlα* mutant using transcription activator-like effector nuclease (TALEN). Two TALENs targeting the *figlα* gene were designed, which resulted in two different alleles (ex1-Δ16 and ex2-Δ4+18) with frameshift mutations causing premature truncations upstream of and at the basic helix-loop-helix domain, respectively (S1G and S1H Fig). At the hatching stage, the number of germ cells was not significantly different between XX wild-type and XX *figlα* homozygous (–/–) gonads (S2A, S2B and S2E Fig), the phenotype of which is consistent with the absence of *figlα* expression in mitotic germ cells at this stage (germ cells in many XX medaka have initiated oogenesis, but do not often reach the pachytene stage). Previously, we identified *foxl3* as a gene involved in the sexual fate decision of germ cells by suppressing the initiation of spermatogenesis in medaka [26]. The expression of FOXL3 is initially observed in both female and male mitotic germ cells (female type I and type II germ cells and male type I germ cells). However, in the male mitotic germ cells, the expression of FOXL3 decreases dramatically by the time of hatching. When *foxl3* function is lost in germ cells of female gonads, spermatogenesis takes place instead of oogenesis by 30dph (after this stage, oogenesis is also initiated by unknown mechanisms). Hence, FOXL3 can be a good marker representing the female sexual fate of germ cells during early gonadal development (from hatching stage to 30dph). In *figlα*–/–mutant gonads, the expression of FOXL3 was found to be normal at hatching stage, which suggests that FOXL3 expression is independently regulated by *figlα* activity. (S2A, S2B and S2F Fig). At 10 dph, XX wild-type (*figlα*+/+) fish had plenty of oocytes at the diplotene stage and started to form follicles (Fig 1A). However, XX *figlα* homozygous (–/–) mutants had cystic germ cells that had arrested between the zygotene and pachytene stages and lacked isolated oocytes at diplotene stage (Fig 1B). The follicle-marker genes, *gdf9* and *bmp15*, were expressed in the oocytes of follicles found in the control ovaries but were not expressed in *figlα*–/–mutant ovaries (Fig 1C–1H). These observations indicate that XX *figlα*–/–mutants did not form follicles.

As in wild-type males, the germ cell proliferation in XY *figlα*–/–mutants was suppressed at hatching (S2C–S2E Fig). XY *figlα*–/–mutants developed normally as fertile males with testis in the adult stage (S3A–S3D Fig). In the efferent ducts, matured sperm were observed (S3E and S3F Fig), suggesting that spermatogenesis was completed.

Next, we examined whether XX *figlα*–/–mutants developed as females. During gonadal development, XX *figlα*–/–mutants expressed the female marker genes *aromatase* and *foxl2*, whereas the male marker, *dmrt1*, was hardly detected (S4 Fig). In the adult stage, female secondary sex characteristics were present, and XX *figlα*–/–mutants had typical ovarian-like gonads with an ovarian cavity, but no follicle formation was observed (Fig 1I–1N). The formation of an ovarian cavity depends on the production of estradiol, which is synthesized by *aromatase* [27]. Accordingly, the adult gonads expressed the *aromatase* as well as *foxl2* (Fig 1O). The expression of these female genes indicates that female somatic cells arise and present in the stromal region of the mutant but cannot contribute to the follicle formation. Taken together, these results demonstrate that normal female development occurs without forming follicles.

**Fig 2:**
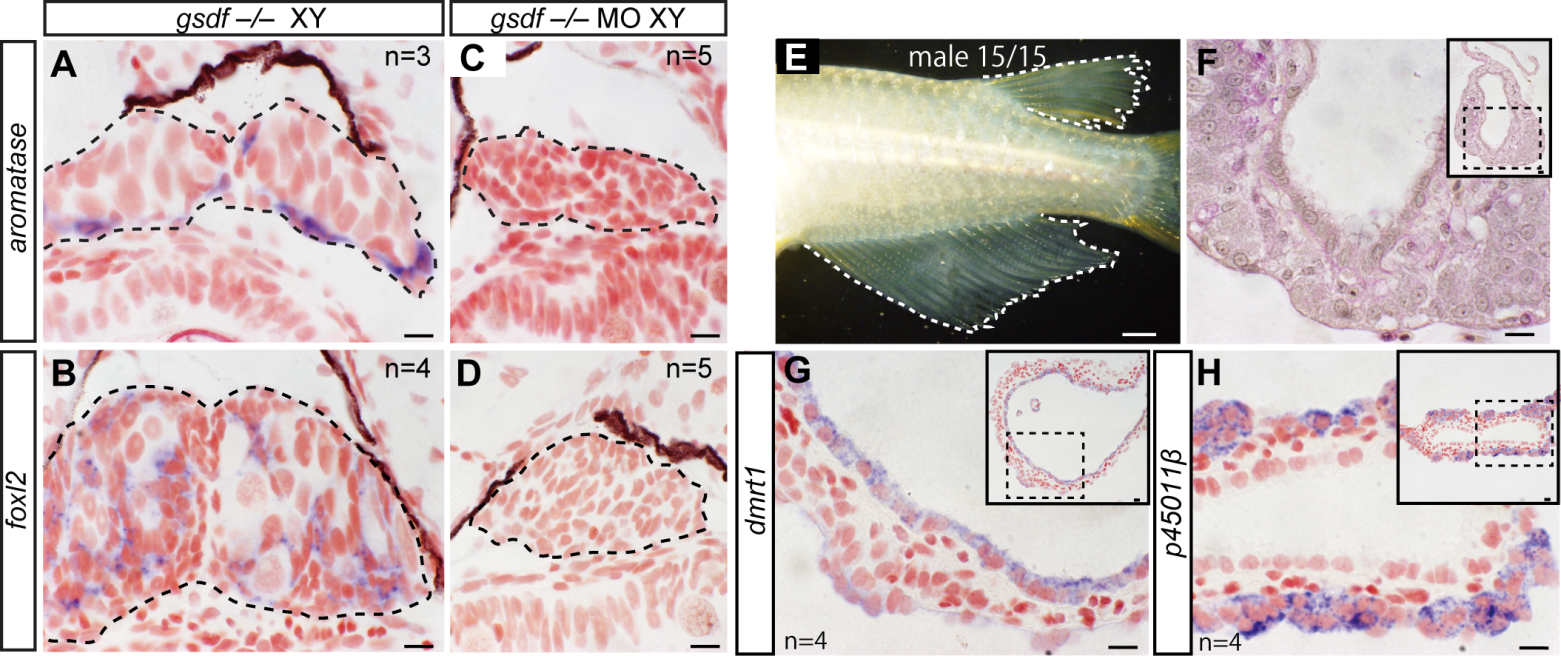
Male-to-female sex reversal in XY *gsdf*–/–mutants is mediated by germ cells. (A–D) *In situ* hybridization for *aromatase* and *foxl2* in *gsdf*–/–XY gonads (10 dph) and germ cell-deficient *gsdf*–/–XY gonads (10 dph) generated by injection of *cxcr4*/*nanos3* morpholino (MO). The female marker genes were detected in *gsdf*–/–XY gonads (A and B, purple signals) but not in germ cell-deficient *gsdf*–/–XY gonads (C and D). The dotted lines indicate the region of gonads. (E) Male secondary sex characteristics of germ cell-deficient adult XY *gsdf*–/–mutants. (F) A cross section of the germ cell-deficient testis-like tubule gonad with PAS staining. (G and H) *In situ* hybridization for *dmrt1* and *p45011β* in the germ cell-deficient gonad of adult XY *gsdf*–/–mutants. *dmrt1* is expressed in the inner side of epithelial cells lining the tubule (G), whereas *p45011β* is expressed in clusters of cells located outside the tubule (H). Dotted boxes in the insets are magnified. Scale bars are 10 μm (A–D, F–H) and 1 mm (E).

In male-to-female sex-reversed medaka, overproliferation of germ cells is associated with follicle formation during gonadal development [12, 13, 19]. To investigate whether follicles are required for male-to-female sex reversal in XY fish, we generated *gsdf* (*g**onadal* *s**oma* *d**erived* *f**actor*) mutants (S5 Fig). *Gsdf* belongs to a TGF-β family that is conserved among teleosts and is enriched in the gonadal somatic cells of males [28, 29]. Although *DMY*/*dmrt1bY* is expressed, XY *gsdf* loss-of-function (*gsdf*–/–) mutants have an increased number of germ cells comparable to that of XX fish at hatching stage, and more than half show male-to-female sex reversal [19, 30]. Consistent with previous studies, our newly generated XY *gsdf*–/–mutants (S5A and S5B Fig) had gonads similar to those of XX fish, and follicle formation occurred during gonadal development (S5C–S5E Fig). XY *gsdf*–/–gonads expressed the female marker genes *foxl2* and *aromatase* at 10 dph (Fig 2A and 2B), whereas germ cell-deficient XY *gsdf*–/–gonads did not express these female markers (Fig 2C and 2D). In the adult stage, the male-to-female sex-reversal was suppressed in XY *gsdf*–/–medaka mutants that had lacked germ cells during the embryonic stage (Fig 2E–2H). These results indicate that the male-to-female sex reversal in XY *gsdf*–/–mutants is mediated by germ cells but not by somatic cell-autonomous feminization.

**Fig 3:**
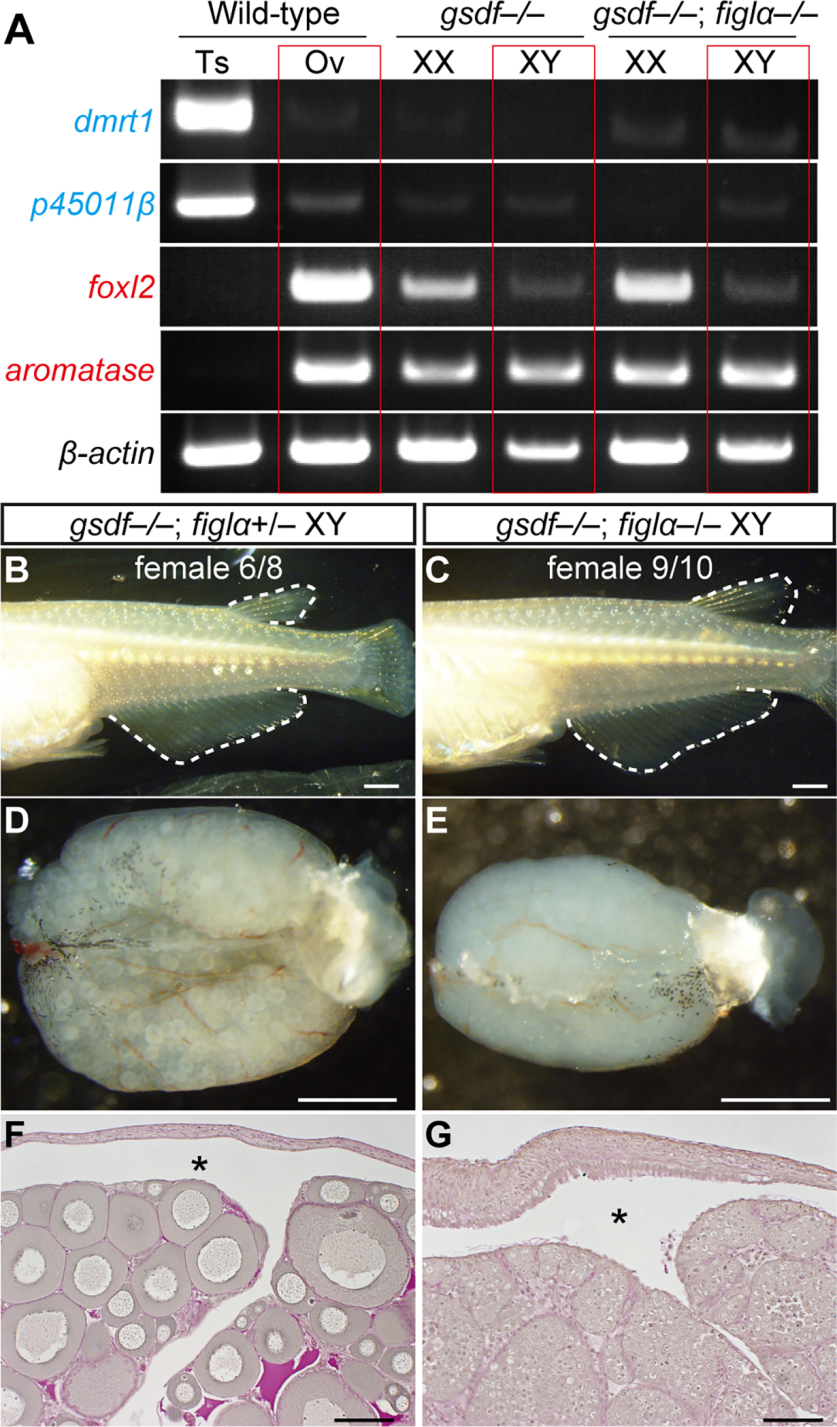
Follicles are not required for male-to-female sex reversal. (A) RT-PCR analysis of male markers, *dmrt1* and *p45011β*, and female markers, *foxl2* and *aromatase*, in adult gonads (4-month-old). The expression pattern of *gsdf*–/–and *gsdf*–/–; *figlα*–/–XY gonads from females is similar to that of wild-type ovaries. (B–C) The secondary sex characteristics of medaka are indicated by the shape of the dorsal and anal fins. Six of eight (6/8) XY *gsdf*–/–; *figlα*+/–and nine of 10 (9/10) XY *gsdf*–/–; *figlα*–/–mutants were female. (D–E) External appearance of the control *gsdf*–/–; *figlα*+/–XY ovary (D) and the *gsdf*–/–; *figlα*–/–XY gonad (E). (F–G) Cross sections of the mutant gonads with PAS staining. Asterisks indicate the ovarian cavity, representing the structure of ovaries. Scale bars are 1 mm (B–E) and 100 μm (F and G).

To determine whether feminization of gonads in *gsdf* mutants requires follicles, we generated *gsdf–/–*; *figlα–/–*double mutants. During gonadal development, XY *gsdf–/–*; *figlα–/–*double mutants had larger gonads with many germ cells compared with those in XY *gsdf*+/–; *figlα*+/–medaka (S6A, S6C, S6D and S6F Fig). Interestingly, the XY double mutants did not form any follicles but expressed *foxl2* and *aromatase*, suggesting that follicles are not required for activation of the feminizing genes (S6 Fig). In the adult stage, nine out of ten XY *gsdf–/–*; *figlα–/–*double mutants exhibited female secondary sex characteristics and had gonads with an ovarian structure and female gene expression (Fig 3). These results may suggest that follicles are not required for male-to-female sex reversal.

### Germ cells prior to gametogenesis are sufficient for feminization

**Fig 4:**
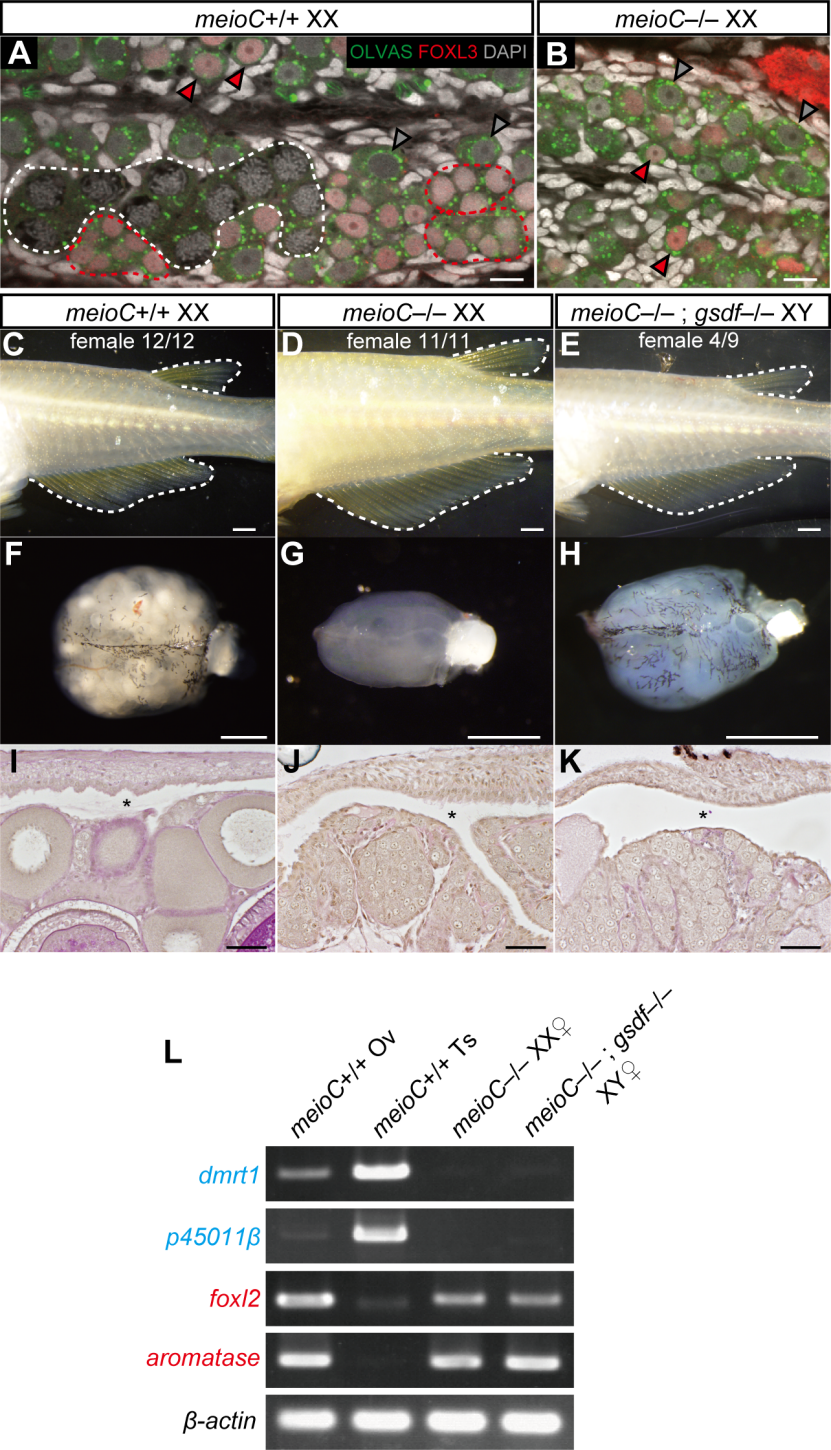
Germ cells before the stage of meiosis are sufficient for feminization of gonads. (A) *meioC*+/+ XX gonads at 5 dph. The gray arrowhead indicates a FOXL3-negative type I germ cell. Red arrowhead indicates FOXL3-positive type I germ cells. The red dotted line indicates FOXL3-positive type II germ cells. The white dotted line indicates meiotic germ cells. (B) *meioC*–/–XX gonad at 5 dph. Only FOXL3-negative (gray arrowhead) and -positive (red arrowhead) type I germ cells are present. (C–E) The secondary sex characteristics of medaka are defined by the shape of dorsal and anal fins. Twelve of 12 (12/12) *meioC*+/+ XX, 11/11 *meioC*–/–XX, and 4/9 *meioC*–/–; *gsdf*–/–XY were female. (F–H) External appearance of the control *meioC*+/+ XX ovary (F), *meioC*–/–XX gonad (G), and *meioC*–/–; *gsdf*–/–XY gonad (H). (I–K) Cross sections of the mutant gonads with PAS staining. Asterisks indicate the ovarian cavity, representing the structure of ovaries. (L) RT-PCR analysis of male markers, *dmrt1* and *p45011β*, and female markers, *foxl2* and *aromatase*, in adult gonads (4-month-old). Gonads from *meioC*–/–XX females and *meioC*–/–; *gsdf*–/–XY females were used. Scale bars are 10 μm (A and B), 1 mm (C–H), and 50 μm (I–K).

We then examined whether germ cells prior to meiosis have a feminizing effect on gonadal somatic cells. Recently, *MeioC* was identified as a gene essential for progression of meiosis [31, 32]. The medaka homologue of *meioC* was predicted from the genomic database (Ensembl ID: ENSORLT0000005275, NCBI ID: XM_011477965.1). It showed conserved synteny among vertebrates (S7A Fig), and it was expressed in the germ cells of both testes and ovaries (S7B and S7C Fig). A medaka mutant was generated using CRISPR/Cas9, resulting in three different alleles with frameshift mutations causing premature truncations upstream of the C-terminal conserved region of the MEIOC protein (S7D and S7E Fig). In 5 dph gonads, XX *meioC* +/+ controls had cystic germ cells and meiotic germ cells (Fig 4A), whereas XX *meioC*–/–mutants had only type I stem-like germ cells in isolation and surrounded by somatic cells. Type II, meiotic germ cells and follicles were not observed (Fig 4B). This suggested that *meioC* is required for the transition of germ cells from type I to type II in medaka. Interestingly, FOXL3 protein was expressed in the XX *meioC*–/–mutants. These results indicate that MEIOC is required for the initiation of gametogenesis, but not for the expression of FOXL3, suggesting that the sexual fate decision of germ cells likely occurs in XX *meioC*–/–gonads. In the adult stage, XX *meioC*–/–mutants showed female secondary characteristics and had gonads with ovarian structures (Fig 4D, 4G and 4J). Furthermore, four of nine XY *meioC–/–*; *gsdf–/–*double mutants also showed female secondary characteristics and had ovary-like gonads (Fig 4E, 4H and 4K) with the expression of *foxl2* and *aromatase* (Fig 4L). Therefore, type II and meiotic germ cells are not required for normal female development in XX fish and male-to-female sex reversal in XY fish.

### PGC-like germ cells have a feminizing effect on somatic cells

**Fig 5:**
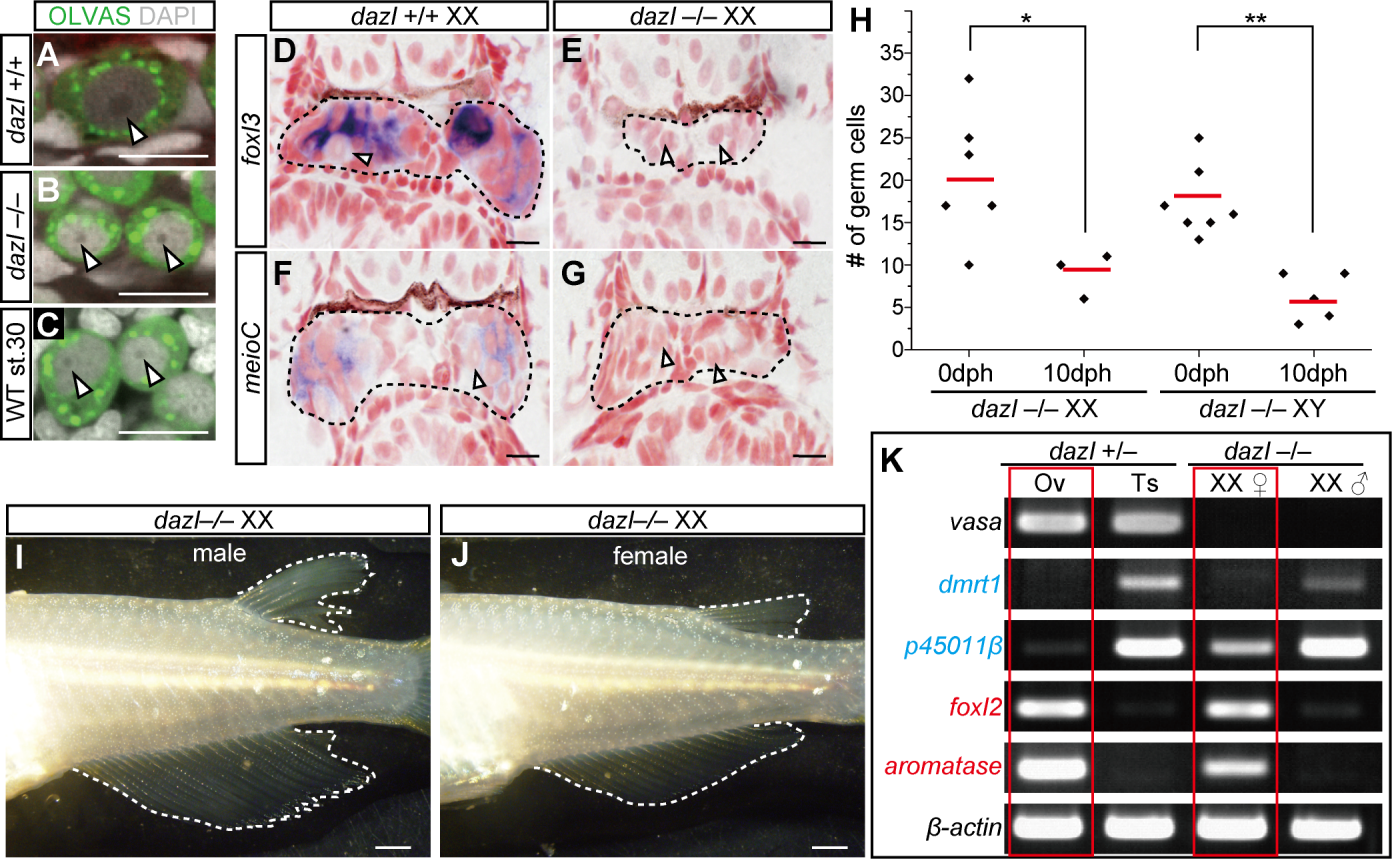
PGC-like germ cells in *dazl* mutants have the potential for feminization of gonads. (A–C) Morphology of type I germ cells observed by immunohistochemistry with OLVAS (green: germ cell) and DAPI staining (gray). Arrowheads indicate nucleoli. *dazl*–/–germ cells at hatching stage (B) are morphologically similar to wild-type PGCs at stage 30 (C) in terms of the small cell-size and a nucleolus size compared to that of wild-type type I germ cells at hatching stage (A). (D–G) *In situ* hybridization of *foxl3* and *meioC* in *dazl*–/–XX gonads at hatching stage. Arrowheads indicate *foxl3* or *meioC* negative germ cells. A black dotted line indicates the region of gonads. (H) The number of germ cells in *dazl*–/–XX and XY gonads at hatching stage (0 dph) and 10 days post hatching (10 dph). * *p*<0.05, **p<0.01 by *t*-test. (I and J) The secondary sex characteristics of medaka are defined by the shape of the dorsal and anal fins. Four of nine (4/9) ex2-Δ34 and 4/12 ex3-Δ2+7 *dazl*–/–XX were male (I), and 5/9 ex2-Δ34 and 8/12 ex3-Δ2+7 *dazl*–/–XX were female (J). (K) RT-PCR analysis of adult gonads in XX *dazl*–/–mutants. The expression pattern of germ cell-deficient gonads from *dazl*–/–XX females was similar to that of *dazl*+/–ovaries. Scale bars are 10 μm (A–G) and 1 mm (H and I).

**Fig 6:**
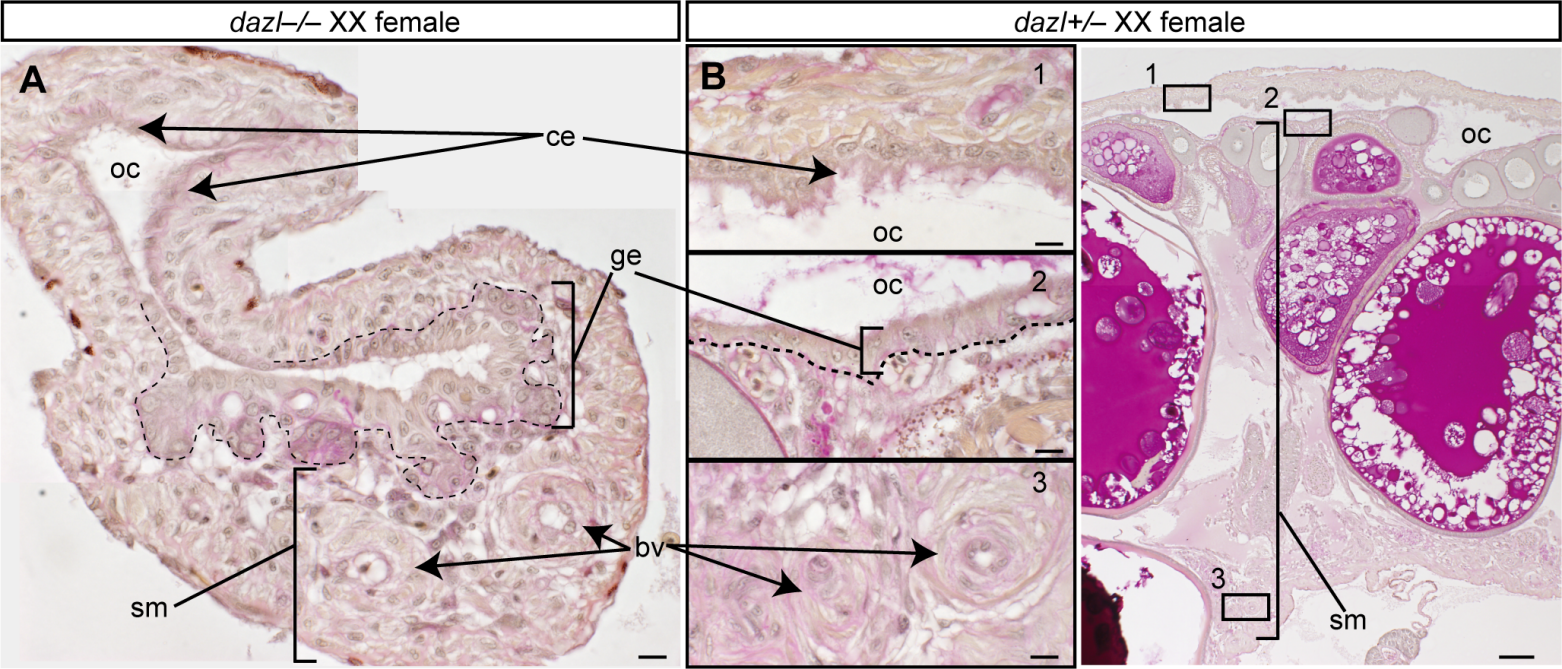
Structure of gonads from *dazl*–/–females. (A–B) Cross sections of *dazl*–/–(A) and *dazl*+/–(B) XX gonads with PAS staining. Boxes with numbers are magnified on the left. The structures of control ovaries consist of the ovarian cavity (oc) and stromal compartment (sm). Between them, the germinal epithelium (ge), where early oogenesis occurs from germline stem cells, is present. In *dazl*–/–female gonads, although germ cells are absent, these ovarian structures are present. bv: blood vessels in the stromal compartment; ce: ciliated epithelial cells lining the ovarian cavity. Scale bars are 10 μm (A, B left) and 100 μm (B right).

Finally, we investigated whether primordial germ cells (PGCs) have the ability to feminize gonads. In mice, *Dazl* is required for germ cells to become gametogenesis-competent, and germ cells in the *Dazl* mutant remain in an undifferentiated state similar to that of PGCs [3]. Therefore, we generated medaka *dazl* mutants using the CRISPR/Cas9 system and obtained two different alleles (ex2-Δ34 and ex3-Δ2+7) that caused premature truncations upstream of or at the RNA recognition motif of DAZL (S8 Fig). Consistent with studies in mice, *dazl*–/–germ cells at hatching stage were morphologically similar to PGCs at st.30, which were characterized by smaller cells and nucleoli than those in *dazl*+/+ type I germ cells at the same stage (Fig 5A–5C). The *foxl3* and *meioC* transcripts were not detected in *dazl*–/–germ cells (Fig 5D–5G), and the number of germ cells was decreased in both XX and XY mutant gonads during gonadal development (Fig 5H). In the adult stage, *vasa* transcripts, a typical marker of germ cells, were not detected in *dazl*–/–gonads (Fig 5K). These results suggest that germ cells in *dazl*–/–mutants lose both competence for gametogenesis and the response to sexual cues from gonadal somatic cells. These germ cells seemed to be no longer maintained in the mutants. In fact, germ cells were absent in the adult stage. Interestingly, not all XX *dazl*–/–mutants developed as males. More than half of the XX mutants (56% in ex2-Δ34 and 67% in ex3-Δ2+7) showed female secondary characteristics (Fig 5I and 5J). The gonads from the phenotypically female XX mutants expressed female genes (Fig 5K). Furthermore, detailed histological analysis revealed that the *dazl*–/–gonads from females had ovarian cavities lined by ciliated epithelial cells, germinal epithelia, and stromal compartments, which are typical structures of ovaries (Fig 6). Therefore, PGC-like germ cells can support female gonadal development.

## Discussion

Germ cells are the only cells that develop into eggs or sperm and produce the next generation. This role has long been recognized as the only function of germ cells. However, recent analyses in teleost fish unveiled another essential role, namely sex determination [11–13, 33, 34]. During normal development in medaka, the first manifestation of sexual dimorphism is the difference in the number of germ cells [9]. However, this difference is not simply a consequence of the direction of sex determination, but rather it is essential for sex determination of the gonad: a greater number of germ cells during gonadal development in XX medaka enables to canalize feminization of gonads, while a lower number of germ cells in XY medaka is not sufficient for feminization, leading to masculinization. Interestingly, manipulating the number of germ cells causes sex-reversal. XY medaka with a greater number of germ cells develop into females, whereas a lower number of germ cells cause XX medaka to differentiate into males [11–13, 35]. A similar relationship between germ cell number during gonadal development and sexual phenotype in the adult stage has also been reported in zebrafish [36]. In this study, we generated four different medaka mutants to demonstrate that the feminizing effect of germ cells is not a result of the progression of gametogenesis or a sexual fate decision of germ cells.

The *dazl–/–*mutants provided insight into the feminizing effect. Approximately half of the XX mutants showed a female phenotype with a normal ovarian structure devoid of germ cells. This phenotype is in sharp contrast with previously reported germ cell-deficient XX medaka, which displays female to male sex-reversal [11]. The difference between the two phenotypes may be explained by the existence of a limited time window during which the feminizing effect of germ cells acts on somatic cells to induce female differentiation. The *dazl–/–*mutants retained a certain number of germ cells during the period in which medaka with female-to-male reversal have a fewer number of germ cells. After this window, the gonad can continue to develop into an ovary even if germ cells are gradually lost in the *dazl* mutants.

The *dazl* mutants also suggest the timing at which germ cells exert a feminizing effect. Our analyses indicate that the germ cells in the *dazl* mutants are likely gametogenesis-incompetent PGCs because they possess PGC-like morphology in both appearance and size and do not express the meiotic gene (*meioC*) or the gene involved in the sexual fate decision of germ cells (*foxl3*). This is consistent with the function of *dazl* in mammals [3]. However, gametogenesis-incompetent germ cells retain the ability to feminize the gonad. This observation suggests that germ cells acquire this property by the time PGCs develop into gonocytes.

The point mentioned above provides insight into the molecular mechanism of gametogenesis. Our results suggest that several mechanisms operate in parallel in developing gametes. Although feminizing effect of germ cells is critical for gonadal sex determination, the mechanism underlying this effect is likely distinct from the sexual fate of germ cells because the feminizing activity is apparent even if *foxl3* is not expressed in the *dazl* mutant. This notion is supported by our previous report that the XX *foxl3* mutant, in which germ cells are committed to spermatogenesis, develops sperm-producing ovaries [26].

In the absence of *meioC* function, germ cells did not undergo cystic division (type II division), demonstrating that *meioC* is critical for the process of transition from type I stem-type germ cells to gametogenesis-committed type II germ cells. The function of the medaka *meioC* is in contrast with that of the mammalian *MeioC*, which is required for the proper progression of meiotic prophase I, but not for the entry of meiosis [31, 32]. Medaka *meioC* may involve more aspects of gametogenesis than mammalian *MeioC*. Both XX *meioC–/–*mutants and XY *gsdf*–/–; *meioC*–/–double mutants possess gonads with typical ovarian structures, indicating that the feminizing effect is manifested before germ cells exit from the status of stem cells/gonocytes and enter the gametogenetic program.

The feminizing effects of germ cells have also been reported in zebrafish [20, 32, 33, 36]. Recently, an oocyte-derived factor, BMP15, was suggested to be required for the maintenance of feminization in zebrafish [21]. On the other hand, in mice and some species of teleost fish such as loach, goldfish and salmon, germ cells are not required for the feminization of gonads [37–41]. These studies provide evidence for the fact that somatic cells can establish and/or maintain feminization without germ cells, but do not deny that feminizing effect of germ cells is absent in these species. In support of this, it is interesting to note that meiotic germ cells in females have antagonizing effects against masculinization in mice [42].

One common feature of germ cells during gonadal sex differentiation in vertebrates is that female germ cells proliferate and initiate oogenesis in an early stage, while male germ cells undergo a mitotically quiescent state before the initiation of spermatogenesis [4, 5]. Why do male germ cells need to be quiescent? Why do they not initiate spermatogenesis with the same timing of oogenesis? Our analyses using medaka collectively suggest that germ cells acquire the feminizing effect before committing to gametogenesis, entering meiosis and the sexual fate decision of germ cells. Therefore, germ cells in medaka may be developmentally designed to make the gonad female. One possible answer for the biological meaning of the quiescent state of male germ cells may be to prevent the gonad from being feminized until masculinization of somatic cells is established.

## Materials and methods

### Ethics statement

All experiments were done according to Regulations on Animal Experiments in Nagoya University. The experimental plan using the small teleost fish is approved by the Nagoya University official ethics committee (Approval Number 11).

### Fish

The OKcab strain and *sox9b*-DsRed/*olvas-*EGFP transgenic medaka were used in this study [17, 43, 44].

### TALEN-induced mutagenesis

The TALEN target sites for *figlα* and *gsdf* were searched using the TALEN Targeter program (https://tale-nt.cac.cornell.edu/node/add/talen) with the following parameters: spacer length of 15–18 bp, repeat array of 16–18 bp, and upstream base of T only. TALEN assembly was performed following a modified version of the original protocol [45]. TALEN plasmids were linearized by NotI digestion and used as templates for *in vitro* RNA synthesis with the mMESSAGE mMACHINE T7 transcription kit (Thermo Fisher). TALEN mRNAs (250 ng/μl left and right) were injected into one- or two-cell stage embryos. The F0 founders were crossed with *sox9b*-DsRed/*olvas*-EGFP transgenic and non-transgenic medaka. Each mutant allele was identified in F1 adult fish using the primer sets shown in S1 Table and sequence analysis.

### CRISPR/Cas9-induced mutagenesis

CRISPR/Cas9 target sites in *meioC* and *dazl* were searched using CRISPR/Cas9 target online predictor (CCTop, http://crispr.cos.uni-heidelberg.de/) [46]. Selected targets sites are shown in S7 and S8 Figs and S1 Table. A designed pair of oligonucleotides (50 μM each) was annealed in TE buffer by heating to 95°C and cooling slowly to 4°C using a thermal cycler (Biometra). The annealed oligos were ligated into BsaI-HF (New England Biolabs) digested DR274 vectors (Addgene plasmid #42250) [47]. The resulting vector was used as a template to amplify fragments containing T7 and gRNA sequences by PCR followed by gel purification. The PCR primer set is shown in S1 Table. Then, the PCR fragments were used as a template for *in vitro* transcription with the MEGAscript T7 kit (Thermo Fisher) according to the manufacturer’s instructions. The synthesized sgRNAs were purified by ammonium acetate precipitation.

For synthesis of *Cas9* mRNA, the pCS2+hSpCas9 vector [48], a gift from Dr. Kinoshita in Kyoto University, was linearized by NotI digestion, followed by *in vitro* transcription using the mMessage mMachine SP6 kit (Thermo Fisher) according to the manufacturer’s instructions. The synthesized RNA was purified with the RNeasy Mini kit (Qiagen). *Cas9* mRNA (50 ng/μl) and sgRNA (25 ng/μl) were injected into one-cell stage embryos. The F0 founders were crossed with *sox9b*-DsRed/*olvas*-EGFP transgenic and non-transgenic medaka. Each mutant allele was identified in F1 adult fish using the primer sets shown in S1 Table and sequence analysis.

### Genotyping

For genotyping of each mutant allele, amplicons including the mutation sites were amplified by SYBR-KOD (TOYOBO), followed by melting curve analysis using StepOnePlus (Applied Biosystems). The primer sets for each mutant allele are shown in S1 Table.

### Generation of *gsdf*–/–; *figlα*–/–and *gsdf*–/–; *meioC*–/–double mutants

To generate *gsdf*–/–; *figlα*–/–double mutants, XX *gsdf*+/–(ex1-Δ3+11) fish were crossed with XY *figlα*–/–(ex1-Δ16) fish to generate *gsdf*+/–; *figlα*+/–fish, followed by incrossing. Subsequently, XX *gsdf*+/–; *figlα*+/–females and XY *gsdf*+/–; *figlα*–/–males were crossed to generate *gsdf*–/–; *figlα*–/–fish. To generate *gsdf*–/–; *meioC–/–*double mutants, XX *gsdf*+/–(ex1-Δ3+11) females were crossed with XY *meioC*+/–(ex5*-*Δ25) males to generate *gsdf*+/–; *meioC*+/–progeny, followed by incrossing.

### *In situ* hybridization, immunohistochemistry, and histology

Whole-mount *in situ* hybridization and immunohistochemistry were performed as previously described [49, 50]. *Figlα* cDNA was a gift of Dr. Kanamori from Nagoya University. The cDNA clones for *gdf9* (clone name: olova52f19), *bmp15* (clone name: olova58c21), and *foxl3* (clone name: olgi46a18) were obtained from NBRP medaka (http://www.shigen.nig.ac.jp/medaka/). The cDNAs were used as templates to amplify T7-taggd PCR fragments, followed by gel purification. The primer sets used for the amplification are shown in S1 Table. Then, DIG-RNA antisense probes were synthesized using DIG RNA labeling mix with T7 RNA polymerase (Roche). The detection of the FOXL3 protein by immunohistochemistry was described previously [26]. Serum or antibodies specific for the following proteins were used: medaka OLVAS [50] (1:100; rat), FOXL3 [26] (1:100; rabbit) and DsRed (1:100; rabbit; Life Technologies). The secondary reagents used were Alexa Fluor 488– and 568–conjugated antibodies (1:100; Molecular Probes). For PAS staining, whole gonads were fixed in Bouin’s solution, and 4-μm–thick plastic sections were prepared using Technovit 8100 (Heraeus Kulzer). The staining procedures were performed as previously described [51].

### Generation of *figlα*-EGFP transgenic medaka

A bacterial artificial chromosome (BAC) transgenic method that uses homologous recombination was employed to generate a *figlα*-EGFP reporter construct as previously described [52]. Specifically, the targeting DNA fragment for recombination was prepared to include the *figlα***-**3′UTR downstream of the EGFP open reading frame. After homologous recombination, this fragment was inserted immediately downstream of the translation initiation site of the *figlα* gene in fosmid clone GOLWFno462_j06. Microinjection of fosmid clone containing EGFP was performed as previously described [52].

### RT-PCR

Total RNA was extracted from adult gonads of wild-type, *figlα*–/–, *gsdf–/–*, *gsdf*–/–; *figlα*+/–, *gsdf*–/–; *figlα*–/–, *moto*–/–, *gsdf*–/–; *moto*–/–, *dazl*+/–, and *dazl*–/–medaka using the TriPure Isolation Reagent (Roche). Two testes and one ovary were used in each experiment. For isolation of RNA from *dazl*–/–medaka, three to five gonads were used. cDNA was produced from 1 μg total RNA using ReverTra Ace (TOYOBO) or SuperScript III (Thermo Fisher) and used as the template for RT-PCR. PCR conditions and primer sets for *vasa*, *dmrt1*, *foxl2*, *p45011β*, *aromatase*, and *β-actin* were described previously [11]. Two independent experiments were performed using separate pools of gonads.

## Supporting information

S1 Fig Expression and TALEN design of *figlα*. (A-D) *In situ* hybridization for *figlα*. *Figlα* transcripts (purple signal) were detected in XX but not in XY gonads at 10 dph (A and B) and in the adult stage (C and D). (E) *figlα*-EGFP reporter medaka in which EGFP is expressed under control of *figlα* regulatory elements, including the promoter and the 3’UTR. (F) Expression of *figlα*-EGFP. EGFP signals (green) were detected in germ cells from the late zygotene stage onward (pachytene and dipotene), but not in mitotic and early meiotic germ cells (type I, type II and early zygotene). VASA granules representing germ cells are visualized as orange. (G) Structure of the *figlα* gene in the medaka genome, nucleotide sequences of TALEN target sites (green), and the resulting deletion and/or insertion (red characters). Deletion of 16 bp upstream of the bHLH domain (ex1-Δ16) and deletion of 4 bp and insertion of 18 bp (ex2-Δ4+18) at the bHLH domain were obtained. (H) Predicted amino-acid sequences of ex1-Δ16 and ex2-Δ4+18 alleles. The orange box indicates the bHLH domain. Black boxes indicate identical amino acids. Scale bars are 10μm.
(TIF)

S2 Fig Proliferation of germ cells is normal in *figlα*–/–gonads at the hatching stage. (A-D) *figlα*+/+ and *figlα*–/–gonads at the hatching stage (7 dpf) observed by immunohistochemistry with OLVAS (green: germ cell), FOXL3 (red) and DAPI staining (gray). Red arrowheads indicate FOXL3-positive type I germ cells. Red dotted lines encircle FOXL3-positive type II germ cells. Scale bars are 10μm. (E) The total number germ cells. (F) The number of FOXL3-positve germ cells. * *p* < 0.05, **p < 0.01 by *t*-test.
(TIF)

S3 Fig XY *figlα–*/–mutants normally develop as males. (A and B) The secondary sex characteristics of medaka are indicated by the shape of the dorsal and anal fins. All observed XY fish were males. (C and D) External appearance of the control *figlα*+/+ XY testis (C) and the *figlα*–/–XY testis (D). (E–F) Cross sections of the testes with PAS staining. Black dotted lines encircle the matured sperm in efferent ducts (ED). Scale bars are 1 mm (A–D) and 50 μm (E and F).
(TIF)

S4 Fig Expression of *aromatase* and *foxl2* without follicles in *figlα*–/–XX gonads. (A–I) *In situ* hybridization for *aromatase*, *foxl2*, and *dmrt1* in *figlα*+/–XX/XY and *figlα*–/–XX gonads at 7 dph. In *figlα*+/–XY gonads, the female markers *aromatase* (A) and *foxl2* (D) were not detected, whereas the male maker *dmrt1* (G) was detected (purple signals). In both *figlα*+/–and *figlα*–/–XX gonads, *aromatase* (B and C) and *foxl2* (E and F) were detected (purple signals), whereas *dmrt1* was hardly detected (H and I). The gonad is encircled by black dotted lines. Scale bars are 10 μm.
(TIF)

S5 Fig TALEN design of *gsdf*. (A) Structure of the *gsdf* gene in the medaka genome, nucleotide sequences of TALEN target sites (green), and the resulting deletion and/or insertion (red characters). Deletion of 7 bp (ex1-Δ7) upstream of the TGF-β domain and deletion of 3 bp and insertion of 11 bp (ex1-Δ3+11) upstream of the TGF-β domain were obtained. (B) Predicted amino-acid sequences of the ex1-Δ7 and ex2-Δ3+11 alleles. The orange box indicates the TGF-β domain. (C–E) Immunohistochemistry of wild-type XX (C), XY (D), and *gsdf*–/–XY gonads (E) at 10 dph. Green: OLVAS (germ cells), gray: DAPI (nucleus). In the wild-type XY gonad (D), only type I germ cells are present. In *gsdf*–/–XY gonads (E), many follicles fill the gonad, which is similar to the wild-type XX gonad (C). Scale bars are 10 μm.
(TIF)

S6 Fig Activation of *aromatase* and *foxl2* without follicles in *gsdf–/–*; *figlα–/–*XY gonads. (A–F) *In situ* hybridization for *aromatase* and *foxl2* in *gsdf+/–*; *figlα+/–*, *gsdf–/–*; *figlα+/–*, and *gsdf–/–*; *figlα–/–*XY gonads at 7 dph. *Aromatase* and *foxl2* were detected in *gsdf–/–*; *figlα+/–*[with follicles (asterisks), B, and E] and *gsdf–/–*; *figlα–/–*(without follicles, C and F) but not in *gsdf+/–*; *figlα+/–*(A and D) XY gonads. Scale bars are 10 μm.
(TIF)

S7 Fig Syntenic analysis, expression, and gRNA design of *meioC*. (A) Syntenic analysis of *meioC*. (B and C) Expression of *meioC* in the adult ovary (B) and testis (C). The black arrowheads and black dotted line indicate *meioC*-expressing germ cells. White arrowheads indicate *meioC*-negative germ cells. (D) Structure of the *meioC* gene in the medaka genome, nucleotide sequences of CRISPR/Cas9 target sites (green and blue), and the resulting deletion and/or insertion (red characters). Insertion of 1 bp (ex5-+1), deletion of 25 bp (ex5-Δ25) and deletion of 20 bp/insertion of 3 bp (ex5-Δ20+3) were obtained. (E) Predicted amino-acid sequences of ex5-+1, ex5-Δ25 and ex5-Δ20+3 alleles. The red box indicates a conserved domain annotated as pfam15189 in NCBI. Scale bars are 10 μm.
(TIF)

S8 Fig gRNA design of *dazl*. (A) Structure of the *dazl* gene in the medaka genome, nucleotide sequences of CRISPR/Cas9 target sites (green and blue), and the resulting deletion and/or insertion (red characters). The RNA recognition motif (RRM) is highlighted in orange. Deletion of 34 bp in exon 2 (ex2-Δ34) and deletion of 2 bp/insertion of 7 bp in exon 3(ex3-Δ2+7) were obtained. (B) Putative amino acid sequences of ex2-Δ34 and ex3-Δ2+7 alleles. The orange box indicates a RRM domain.
(TIF)

S1 Table Primers used in this study. (XLSX)
