# Supplementary figures and images for: Germ cells in the teleost fish medaka have an inherent feminizing effect
Source: PLoS Genet. 2018 Mar 29;14(3):e1007259. doi: 10.1371/journal.pgen.1007259 (PMC5875746; doi:10.1371/journal.pgen.1007259)

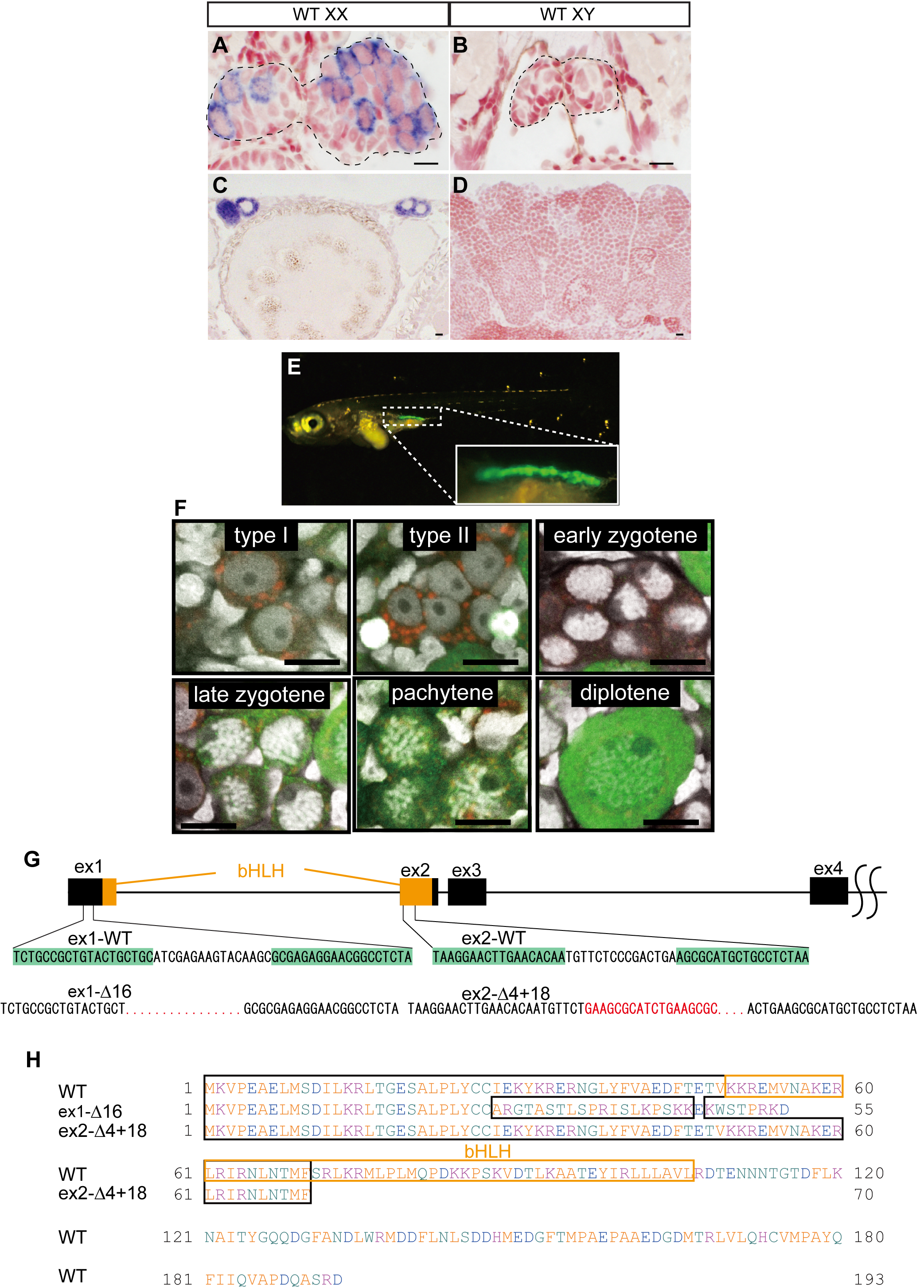

Supplement: S1 Fig — (A-D) In situ hybridization for figlα. Figlα transcripts (purple signal) were detected in XX but not in XY gonads at 10 dph (A and B) and in the adult stage (C and D). (E) figlα-EGFP reporter medaka in which EGFP is expressed under control of figlα regulatory elements, including the promoter and the 3’UTR. (F) Expression of figlα-EGFP. EGFP signals (green) were detected in germ cells from the late zygotene stage onward (pachytene and dipotene), but not in mitotic and early meiotic germ cells (type I, type II and early zygotene). VASA granules representing germ cells are visualized as orange. (G) Structure of the figlα gene in the medaka genome, nucleotide sequences of TALEN target sites (green), and the resulting deletion and/or insertion (red characters). Deletion of 16 bp upstream of the bHLH domain (ex1-Δ16) and deletion of 4 bp and insertion of 18 bp (ex2-Δ4+18) at the bHLH domain were obtained. (H) Predicted amino-acid sequences of ex1-Δ16 and ex2-Δ4+18 alleles. The orange box indicates the bHLH domain. Black boxes indicate identical amino acids. Scale bars are 10μm. (TIF) [file pgen.1007259.s001.tif]

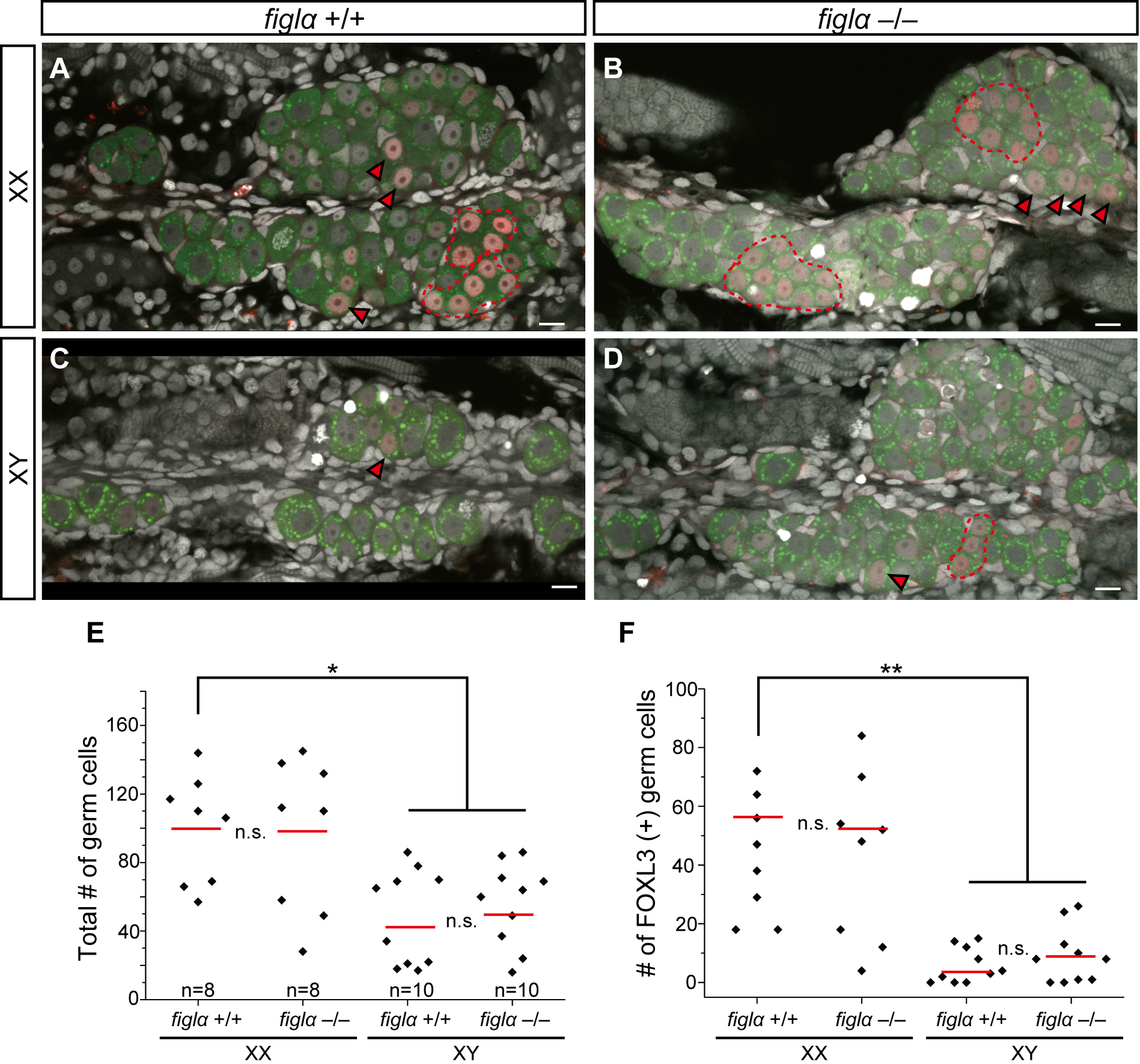

Supplement: S2 Fig — (A-D) figlα+/+ and figlα–/–gonads at the hatching stage (7 dpf) observed by immunohistochemistry with OLVAS (green: germ cell), FOXL3 (red) and DAPI staining (gray). Red arrowheads indicate FOXL3-positive type I germ cells. Red dotted lines encircle FOXL3-positive type II germ cells. Scale bars are 10μm. (E) The total number germ cells. (F) The number of FOXL3-positve germ cells. * p < 0.05, **p < 0.01 by t-test. (TIF) [file pgen.1007259.s002.tif]

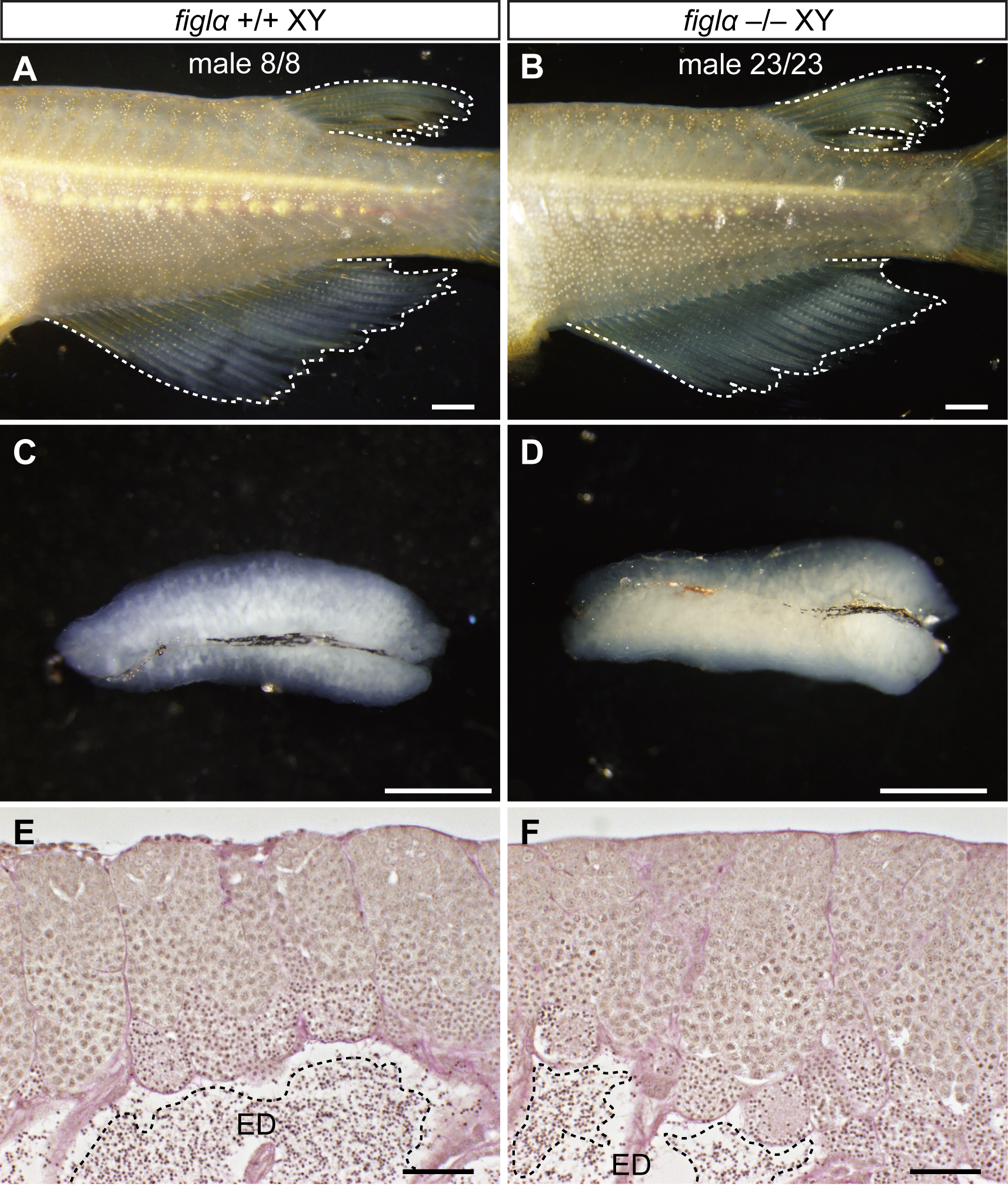

Supplement: S3 Fig — (A and B) The secondary sex characteristics of medaka are indicated by the shape of the dorsal and anal fins. All observed XY fish were males. (C and D) External appearance of the control figlα+/+ XY testis (C) and the figlα–/–XY testis (D). (E–F) Cross sections of the testes with PAS staining. Black dotted lines encircle the matured sperm in efferent ducts (ED). Scale bars are 1 mm (A–D) and 50 μm (E and F). (TIF) [file pgen.1007259.s003.tif]

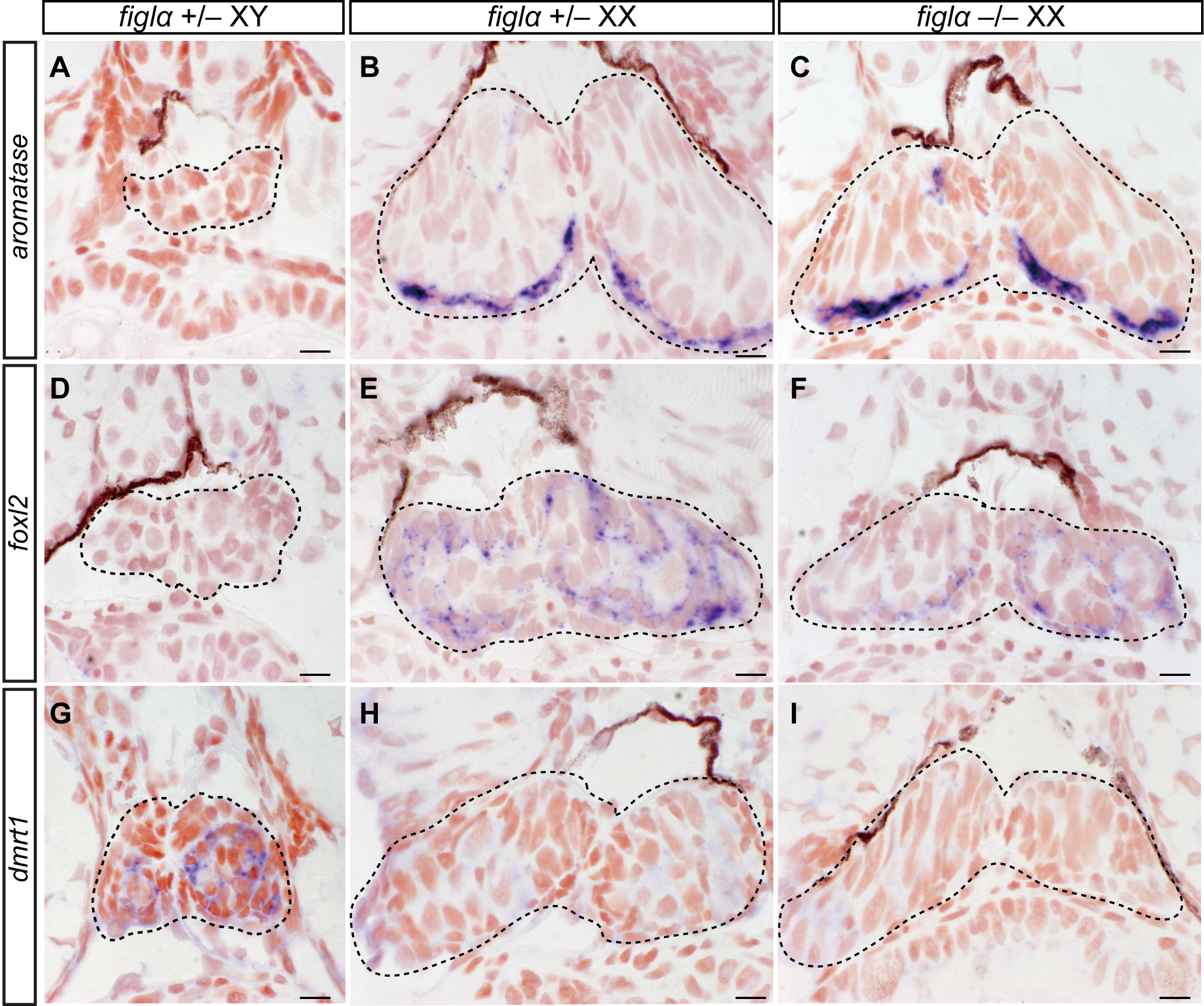

Supplement: S4 Fig — (A–I) In situ hybridization for aromatase, foxl2, and dmrt1 in figlα+/–XX/XY and figlα–/–XX gonads at 7 dph. In figlα+/–XY gonads, the female markers aromatase (A) and foxl2 (D) were not detected, whereas the male maker dmrt1 (G) was detected (purple signals). In both figlα+/–and figlα–/–XX gonads, aromatase (B and C) and foxl2 (E and F) were detected (purple signals), whereas dmrt1 was hardly detected (H and I). The gonad is encircled by black dotted lines. Scale bars are 10 μm. (TIF) [file pgen.1007259.s004.tif]

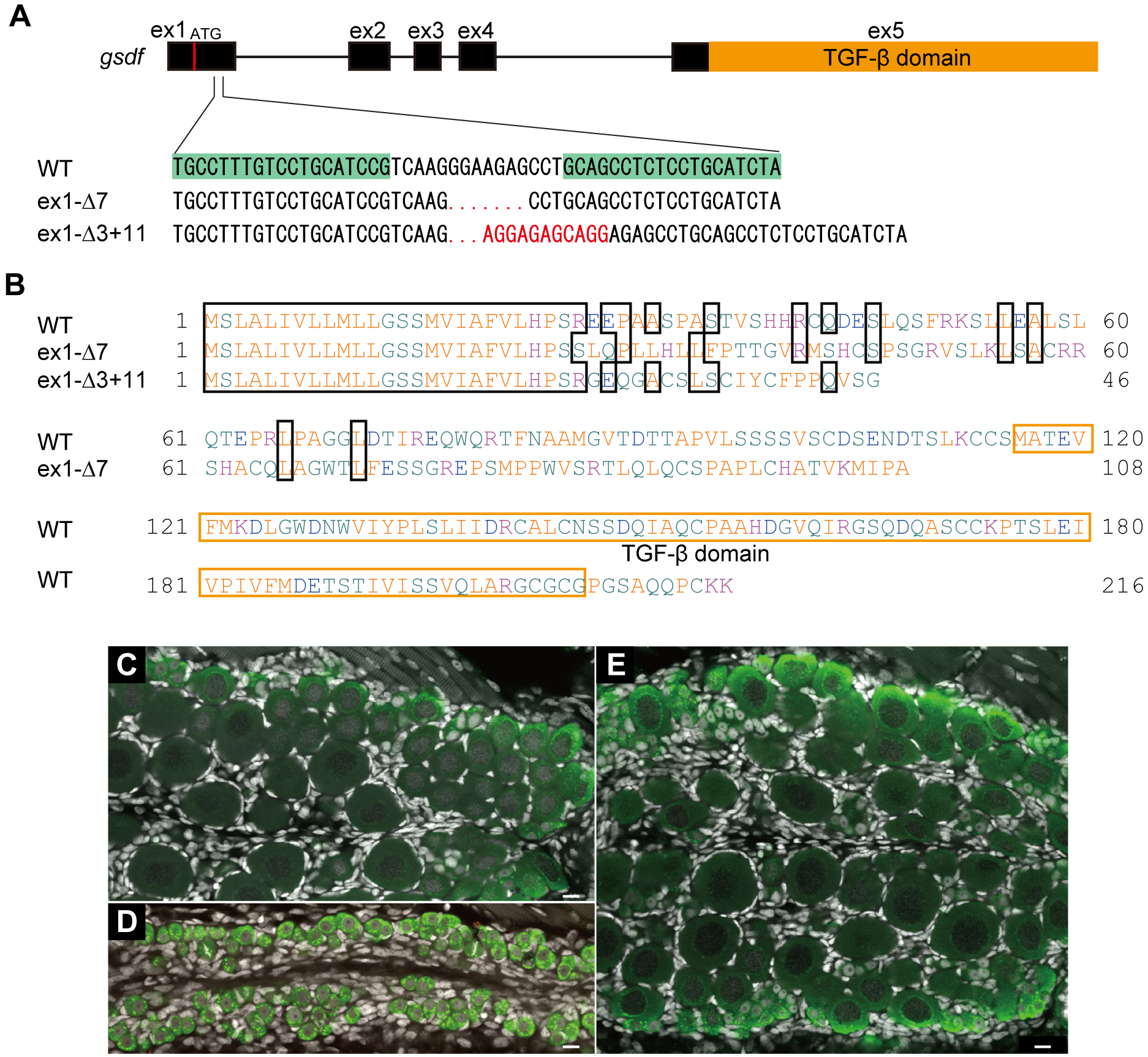

Supplement: S5 Fig — (A) Structure of the gsdf gene in the medaka genome, nucleotide sequences of TALEN target sites (green), and the resulting deletion and/or insertion (red characters). Deletion of 7 bp (ex1-Δ7) upstream of the TGF-β domain and deletion of 3 bp and insertion of 11 bp (ex1-Δ3+11) upstream of the TGF-β domain were obtained. (B) Predicted amino-acid sequences of the ex1-Δ7 and ex2-Δ3+11 alleles. The orange box indicates the TGF-β domain. (C–E) Immunohistochemistry of wild-type XX (C), XY (D), and gsdf–/–XY gonads (E) at 10 dph. Green: OLVAS (germ cells), gray: DAPI (nucleus). In the wild-type XY gonad (D), only type I germ cells are present. In gsdf–/–XY gonads (E), many follicles fill the gonad, which is similar to the wild-type XX gonad (C). Scale bars are 10 μm. (TIF) [file pgen.1007259.s005.tif]

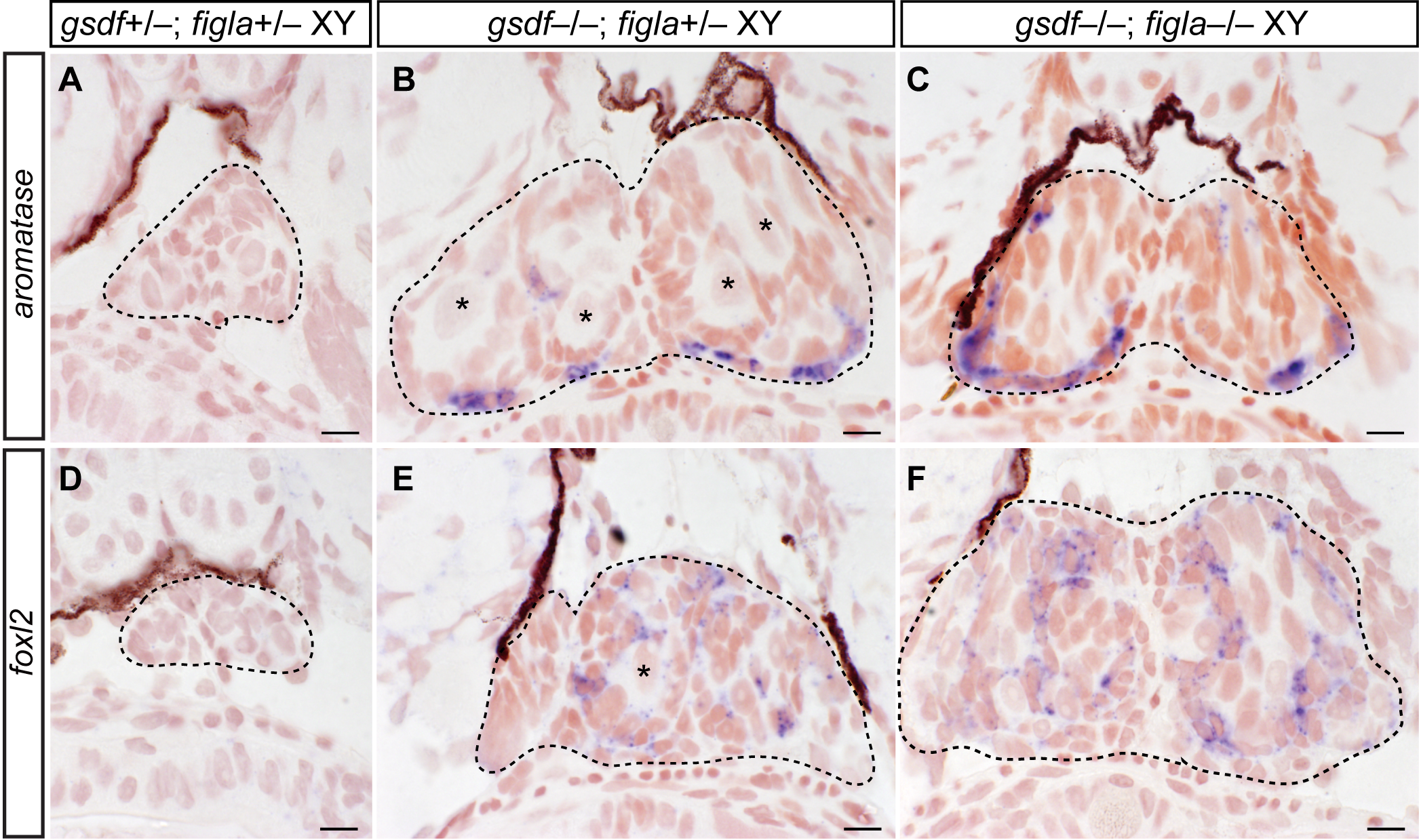

Supplement: S6 Fig — (A–F) In situ hybridization for aromatase and foxl2 in gsdf+/–; figlα+/–, gsdf–/–; figlα+/–, and gsdf–/–; figlα–/–XY gonads at 7 dph. Aromatase and foxl2 were detected in gsdf–/–; figlα+/–[with follicles (asterisks), B, and E] and gsdf–/–; figlα–/–(without follicles, C and F) but not in gsdf+/–; figlα+/–(A and D) XY gonads. Scale bars are 10 μm. (TIF) [file pgen.1007259.s006.tif]

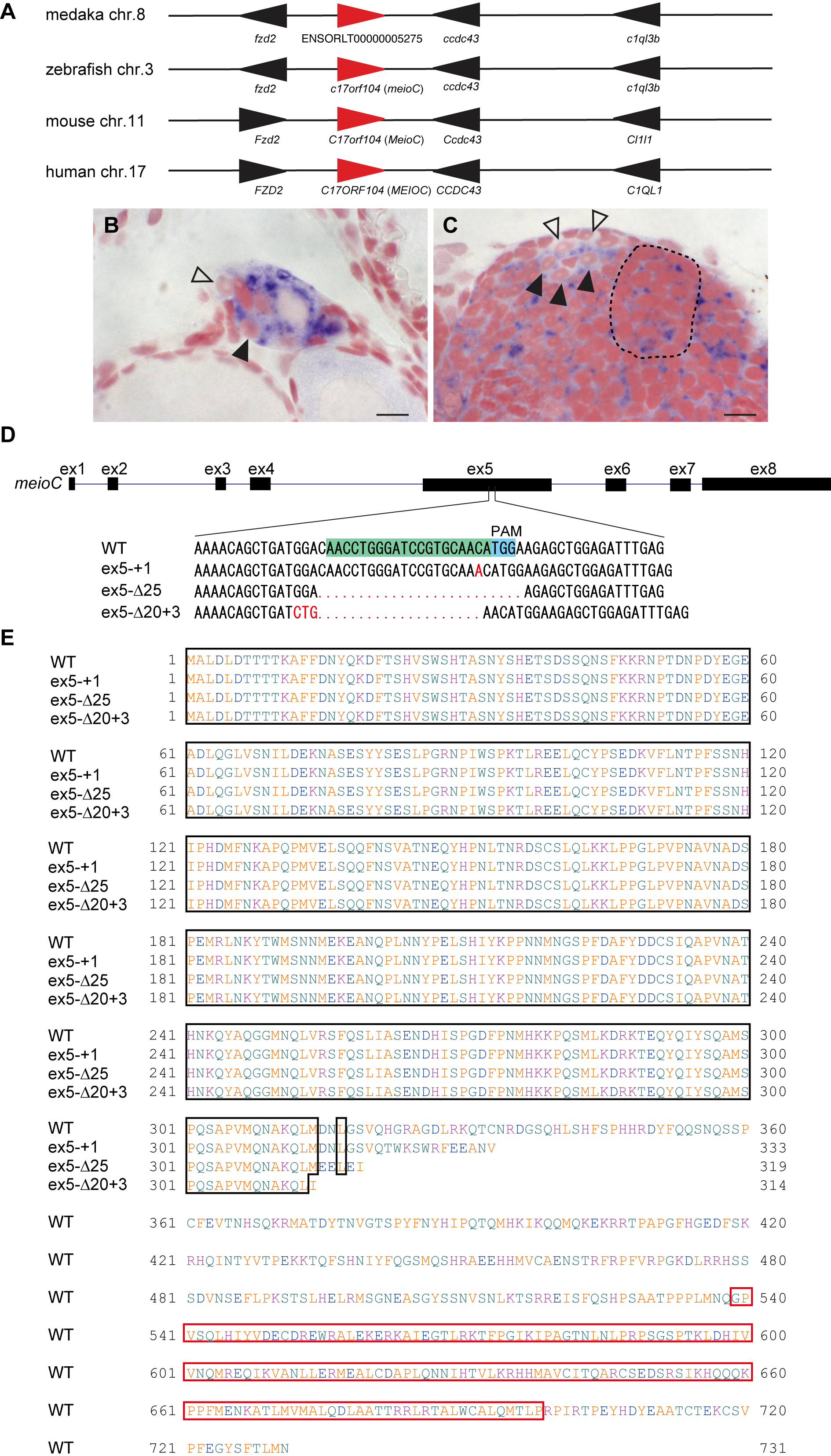

Supplement: S7 Fig — (A) Syntenic analysis of meioC. (B and C) Expression of meioC in the adult ovary (B) and testis (C). The black arrowheads and black dotted line indicate meioC-expressing germ cells. White arrowheads indicate meioC-negative germ cells. (D) Structure of the meioC gene in the medaka genome, nucleotide sequences of CRISPR/Cas9 target sites (green and blue), and the resulting deletion and/or insertion (red characters). Insertion of 1 bp (ex5-+1), deletion of 25 bp (ex5-Δ25) and deletion of 20 bp/insertion of 3 bp (ex5-Δ20+3) were obtained. (E) Predicted amino-acid sequences of ex5-+1, ex5-Δ25 and ex5-Δ20+3 alleles. The red box indicates a conserved domain annotated as pfam15189 in NCBI. Scale bars are 10 μm. (TIF) [file pgen.1007259.s007.tif]

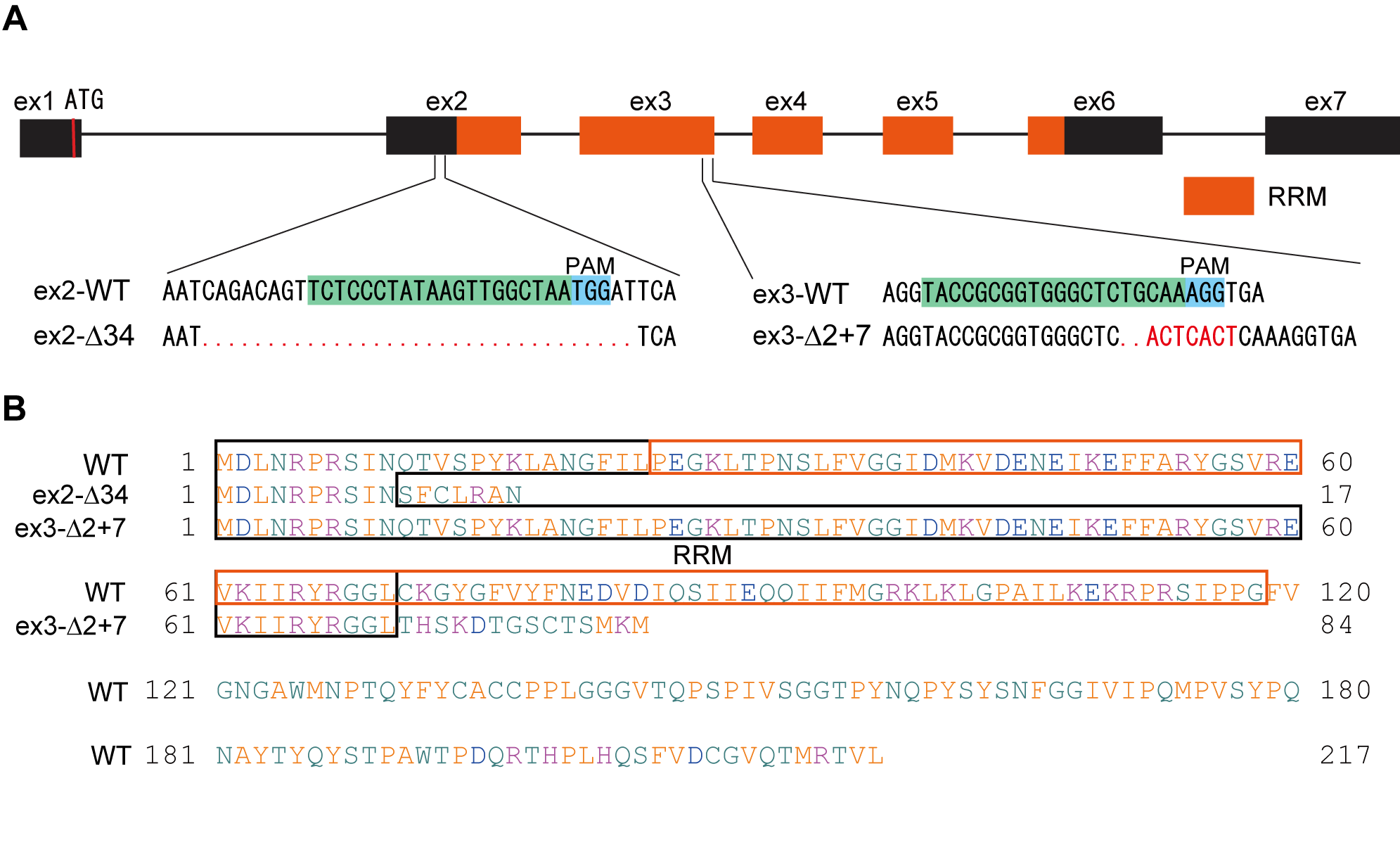

Supplement: S8 Fig — (A) Structure of the dazl gene in the medaka genome, nucleotide sequences of CRISPR/Cas9 target sites (green and blue), and the resulting deletion and/or insertion (red characters). The RNA recognition motif (RRM) is highlighted in orange. Deletion of 34 bp in exon 2 (ex2-Δ34) and deletion of 2 bp/insertion of 7 bp in exon 3(ex3-Δ2+7) were obtained. (B) Putative amino acid sequences of ex2-Δ34 and ex3-Δ2+7 alleles. The orange box indicates a RRM domain. (TIF) [file pgen.1007259.s008.tif]
